# New vectors of Rift Valley fever in West Africa.

**DOI:** 10.3201/eid0402.980218

**Published:** 1998

**Authors:** D Fontenille, M Traore-Lamizana, M Diallo, J Thonnon, J P Digoutte, H G Zeller

**Affiliations:** Laboratoire de zoologie médicale, Institut Pasteur, Dakar, Sénégal. fontenil@dakar.orstom.sn

## Abstract

After an outbreak of Rift Valley fever in Southern Mauritania in 1987, entomologic studies were conducted in a bordering region in Sénégal from 1991 to 1996 to identify the sylvatic vectors of Rift Valley fever virus. The virus was isolated from the floodwater mosquitoes Aedes vexans and Ae. ochraceus. In 1974 and 1983, the virus had been isolated from Ae. dalzieli. Although these vectors differ from the main vectors in East and South Africa, they use the same type of breeding sites and also feed on cattle and sheep. Although enzootic vectors have now been identified in West Africa, the factors causing outbreaks remain unclear.


### New Vectors of Rift Valley Fever in West Africa

**D. Fontenille,* M. Traore-Lamizana,* M. Diallo,*† J. Thonnon,† J.P. Digoutte,† and H.G. Zeller‡**
*Laboratoire de zoologie médicale, Institut Francais de recherche scientifique pour le développement en coopération (ORSTOM), Institut Pasteur, BP 1386, Dakar, Sénégal; †Institut Pasteur, BP 220, Dakar, Sénégal; ‡Institut Pasteur, BP 1274, Antananarivo, Madagascar

After an outbreak of Rift Valley fever in Southern Mauritania in 1987, entomologic studies were conducted in a bordering region in Sénégal from 1991 to 1996 to identify the sylvatic vectors of Rift Valley fever virus. The virus was isolated from the floodwater mosquitoes *Aedes vexans* and *Ae. ochraceus*. In 1974 and 1983, the virus had been isolated from *Ae. dalzieli*. Although these vectors differ from the main vectors in East and South Africa, they use the same type of breeding sites and also feed on cattle and sheep. Although enzootic vectors have now been identified in West Africa, the factors causing outbreaks remain unclear.

Rift Valley fever (RVF) virus belongs to the genus *Phlebovirus,* family Bunyaviridae. RVF virus epizootics, which cause abortions and deaths in young ungulates and epidemics with hemorrhagic fever and other symptoms, have occurred throughout sub-Saharan Africa and Egypt (1). The virus was first isolated in 1930 in Kenya. The most recent outbreaks occurred in Egypt in 1977-1978 and 1993, South Mauritania in 1987, Madagascar in 1990-1991 (2), and Northern Kenya and Somalia in 1997 (3). The virus is transmitted by mosquitoes and by aerosols of viremic blood during hemorrhage.

After the virus was isolated from livestock, humans, and mosquitoes, transmission cycles were proposed for East and South Africa (1). In East and South Africa, the virus is transmitted from floodwater *Aedes* (subgenera *Aedimorphus* and *Neomelaniconion)* to vertebrates or from mosquito to mosquito by vertical transmission. Vectors (*Ae. cumminsii, Ae. circumluteolus,* and *Ae. mcintoshi)* breed in temporary flood ponds, which flood extensively during heavy rainfall. After such rainfall, the number of mosquitoes increases dramatically, and epizootic and epidemic cycles can occur. During epidemics, other mosquito species can serve as vectors; transmission also occurs by aerosol from the blood of viremic vertebrates. Data obtained in the last few years, however, have shown that the pattern of RVF transmission is different in West Africa (4).

Address for correspondence: Didier Fontenille, Laboratoire ORSTOM de zoologie médicale, Institut Pasteur, BP 1386, Dakar, Sénégal; fax: 221 839 92 10; e-mail: fontenil@dakar.orstom.sn.

Table 1. Rift Valley fever virus isolates in West and Central Africa

| Host | No. of isolates | Location | Year(s) |
|---|---|---|---|
| *Aedes dalzieli* | 3 | Kédougou, Sénégal | 1974 |
| *Ae. dalzieli* | 1 | Kédougou, Sénégal | 1983 |
| *Ae. ochraceus* | 3 | Barkedji, Sénégal | 1993 |
| *Ae. vexans* | 10 | Barkedji, Sénégal | 1993 |
| *Ae. cumminsii* | 1 | Burkina-Faso | 1983 |
| *Ae. furcifer* | 1 | Burkina-Faso | 1983 |
| *Culex antennatus* | 1 | Nigeria | 1967-70 |
| *Culicoides sp.* | 2 | Nigeria | 1967 |
| *Ae. palpalis* | 1 | Central African Republic | 1969 |
| *Mansonia africana* | 1 | Central African Republic | 1969 |
| *Amblyomma variegatum* (on cattle in a slaughterhouse) | 1 | Central African Republic | 1983 |
| Humans | 2 | Sénégal | 1975 |
| | 1 | Sénégal | 1980 |
| | 201 | Mauritania | 1987 |
| | 12 | Central African Republic | 1971-90 |
| Bats | 2 | Guinea | 1981-83 |
| Sheep | 1 | Barkedji, Sénégal | 1993 |
| Bovine | 1 | Kolda, Sénégal | 1993 |

Before 1991, RVF virus had been isolated from mosquitoes, humans, and bats in different West African countries (Table 1). Serologic data have demonstrated active transmission of the virus throughout West Africa (5).

In Sénégal, RVF virus was isolated from *Aedes dalzieli* in 1974 and 1983. RVF virus had never been isolated from this species in East and South Africa. The only known RVF outbreak, resulting in more than 200 human deaths, occurred in Southern Mauritania near the village of Rosso, on the Sénégal River (6). Serologic surveys of cattle after the outbreak showed that the epizootic was widespread. Animals with positive immuglobulin (IgM) were recorded in The Gambia 340 km south of the epidemic (7). However, surveys conducted 1 to 2 years after the outbreak showed a decrease in RVF seroprevalence, which suggested that the transmission had ceased (8). No human cases were observed in Sénégal after the outbreak. In 1995 and 1996, IgM-positive sheep and cattle were observed again along the Sénégal River, demonstrating that the virus remains present in the region (Thonnon, unpub. data). Because the virus was not isolated from approximately the 500,000 mosquitoes captured in the Rosso area the year after the outbreak, we decided to identify the mosquitoes or other arthropod species involved in the RVF-endemic cycles. We wanted to determine where the virus was when there were no visible manifestations in humans or cattle and whether the mosquito vectors were the same there as in East Africa.

On the basis of the epidemiology of the RVF virus in East and South Africa and the few isolations from West Africa, we selected for a study of the sylvatic vectors of RVF two sites in different bioclimatic areas in Sénégal: Kédougou, where the virus had been isolated before, and Barkedji, where temporary ground pools occur. We focused on mosquitoes and sand flies because RVF is a phlebovirus and research had shown that *Phlebotomus duboscqi* can transmit the virus (9). This research also first identified in the sahelian region the sylvatic vectors of RVF (10), which are different from those in East and South Africa.

Entomologic surveys were conducted from 1991 to 1996. The Kédougou area (12°ll'N, 12°33'W), in southeastern Sénégal in the Sudano-Guinean bioclimatic zone, has a rainy season (May through October) and an average rainfall of 1,100 mm. The Barkedji area (15°17'N, 14°17'W), in northern Sénégal in the sahelian Ferlo region, has a short rainy season (July to September) with an average rainfall of 250-350 mm. Temporary ground pools fill soon after the first rains and remain the only source of water during the dry season until January.

Hematophagous arthropods were collected each year in July, October, and November in Kédougou, and monthly in Barkedji. Insects were captured by four methods: when they landed to bite human volunteers from 17:30 to 22:30; with Centers for Disease Control and Prevention (CDC)–dry ice light traps; with CDC light traps located in sheepfolds, and with animal-baited (one sheep or three chickens) intermittent light traps. In Kédougou, arthropods were collected in villages and in a forest, and in Barkedji, at the edge of three temporary ground pools.

Arthropods were sorted and pooled by species, sex, location, and date in the field. Pools of arthropods (fewer than 100) were put in liquid nitrogen and stored at -70°C. Viruses were isolated on AP61 *(Ae. pseudoscutellaris)* and Vero cell cultures. Some mosquito pools were injected into suckling mice. Viruses were detected by immunofluorescence assay that used specific mouse immune ascitic fluids (11). Viruses were identified by complement fixation and neutralization tests. Blood-fed mosquitoes collected in the traps were preserved so that the blood meal source could be identified by enzyme-linked immunosorbent assay (ELISA) (12).

More than 228,000 mosquitoes from 52 species in Barkedji and 250,000 mosquitoes from 102 species in Kédougou were collected and tested for virus isolation. Additionally, 233,000 sand flies from 11 species and 35,000 sand flies from 25 species were caught in Barkedji and Kédougou, respectively (Table 2). In Barkedji, *Aedes* species represented 28.8% of the mosquitoes collected. *Ae.(Aedimorphus) vexans* was the most abundant *Aedes* species collected, followed by *Ae.(Adm) ochraceus; Ae. (Neomelaniconion) mcintoshi* and *Ae. (Adm) dalzieli* were rare. Sand flies were abundant during the dry season (December through May). In Kédougou, *Aedes* species represented 50.6% of the mosquitoes collected. *Ae. dalzieli* was the predominant *Aedes* species; *Ae. vexans, Ae. mcintoshi*, and *Ae. ochraceus* were much less abundant.

In Barkedji, 10 RVF virus isolates came from *Ae. vexans* and 3 from *Ae. ochraceus* collected around three temporary ground pools and near

Table 2. Arthropod collections in Kédougou and Barkedji, 1991 to 1996

| Location | Year | Mosquitoes No. | Mosquitoes (Pools) | *Aedes* spp. No. | *Aedes* spp. (Pools) | Sand flies No. | Sand flies (Pools) |
|---|---|---|---|---|---|---|---|
| Barkedji | 1991 | 34,327 | (1,042) | 11,233 | (338) | 3,370 | (56) |
| | 1992 | 42,804 | (1,534) | 5,782 | (352) | 30,547 | (269) |
| | 1993 | 64,810 | (2,023) | 6,056 | (300) | 72,104 | (651) |
| | 1994 | 22,470 | (927) | 8,701 | (365) | 42,730 | (416) |
| | 1995 | 40,952 | (1,413) | 16,124 | (561) | 66,300 | (667) |
| | 1996 | 23,041 | (764) | 16,243 | (453) | 18,934 | (192) |
| Total | | 228,404 | (7,703) | 64,139 | (2,369) | 233,895 | (2,251) |
| Kédougou | 1991 | 48,377 | (1,264) | 28,579 | (730) | 200 | (2) |
| | 1992 | 37,685 | (1,364) | 26,889 | (804) | 1,032 | (9) |
| | 1993 | 71,992 | (2,493) | 31,224 | (1,136) | | |
| | 1994 | 48,751 | (1,877) | 23,839 | (956) | | |
| | 1995 | 17,756 | (1,014) | 12,590 | (616) | 23,122 | (238) |
| | 1996 | 25,768 | (1,284) | 14,869 | (679) | 11,215 | (115) |
| Total | | 250,329 | (9,296) | 126,659 | (4,914) | 35,569 | (364) |
| Total | (1991-96) | 478,733 | (16,999) | 190,798 | (7,283) | 269,464 | (2,615) |

cattle droves in CO2-CDC light traps in October and November 1993. In November, an RFV virus isolate was obtained from one of the sheep. West Nile, Ngari, and Wesselsbron viruses were also isolated from vectors or potential RVF vectors. Five viruses were isolated from sand flies (Table 3).

In Kédougou, no RVF virus was isolated from any vector during the study period, although the virus had been found four times in earlier isolates obtained in 1974 and 1982 from *Ae.dalzieli*. This study found other viruses isolated from *Ae. dalzieli* including 42 viral strains belonging to seven different viruses, mostly alphavirus and flavivirus, one strain of Wesselsbron virus was isolated from *Ae. ochraceus*, and five different viruses were isolated from sand flies (Table 3).

Even if the vectorial competence of *Ae.vexans, Ae. ochraceus*, and *Ae. dalzieli* had not been experimentally confirmed, they would be likely enzootic vectors of RVF virus in Sénégal. These three species are different from the main known East African vectors—*Ae.cumminsii, Ae. circumluteolus*, and *Ae. mcintoshi*—which are also in West Africa, but whose role in RVF virus transmission has not been demonstrated. A much lower number of *Ae. mcintoshi* than *Ae.vexans* or *Ae. ochraceus* were captured each year: when RVF virus was isolated in 1993 in Barkedji, 6,958 *Ae. vexans*, 1,069 *Ae. ochraceus*, but only 58 *Ae. mcintoshi* were captured and tested.

Other Sénégalese floodwater zoophilic mosquitoes such as *Ae.(Adm) minutus, Ae. (Adm) fowleri*, and *Ae. (Adm) argenteopuntatus* should be suspected as potential sylvatic RVF vectors because they belong to the same *Aedes* subgenera of known vectors and have almost the same breeding sites and trophic behavior (10). Moreover, females from a colony of Sénégalese *Ae. fowleri* were able to transmit RVF virus experimentally (13). Mosquito species belonging to other genera *(e.g., Culex, Mansonia)* should be implicated during an outbreak only after the virus is amplified, as in East Africa (3).

The taxonomic status of *Ae. vexans*, the chief enzootic vector of RVF virus, has to be clarified. *Ae. vexans* has a worldwide distribution. In 1975, G.B. White wrote that the African specimens of *Ae. vexans* belong to the same subspecies *Ae. vexans arabiensis*, which was recorded in Mauritania, Sénégal, The Gambia, Ghana, Nigeria, Sudan, Ethiopia, Somalia, and South Africa (14). In Barkedji, in the dry sahelian region of Sénégal, female *Ae. vexans* lay eggs on the soil of temporary ground pools. The adult *Ae. vexans* appear only 4 days after the first rain, a very short larval development period. *Ae. vexans* can be abundant (more than 4,000 per light trap). However, their density decreases quickly about 2 months after the ground pools flood, while the density of other species, such as *Culex poicilipes, Mimomyia splendens*, or *Mansonia africana*, increases at the end of the rainy season (Figure).

Evaluating the trophic preferences of mosquitoes is difficult and highly dependent on the sampling methods. Some species are highly host-specific, but most species feed on a range of different host animals. Despite numerous human bites by *Ae. vexans* and *Ae. ochraceus* around the pools, these species seemed not very host-specific. Females that fed on cattle, sheep, goats, horses, and even chickens were identified. Other sylvatic vectors or potential vectors of RVF virus also showed equally low host specificity (Table 4).

Because of low host specificity, many different viruses, including RVF, can be transmitted to many vertebrate species. More than 30 viruses belonging to different groups have been isolated from *Ae. vexans* throughout the world (15)—the three from West Africa include West Nile virus (a flavivirus from birds) and Ngari virus (isolated from ill humans). Fifteen viruses, also from different arbovirus groups, have been isolated from *Ae. dalzieli* in West Africa (16). Few viruses were isolated from *Ae. ochraceus* and *Ae. mcintoshi*, probably because of the lower number of pools tested.

The risk for a new outbreak of RVF in Sénégal is highly speculative. An increase in IgG prevalence in livestock in different West African countries the year before the 1987 Mauritanian outbreak demonstrated that the virus was present in endemic cycles in this area (17). This epizootic was caused by three factors: a dam was built near Rosso on the Sénégal River, mosquito density increased probably because of the flooding of the river bank in 1987, and the livestock density increased. In 1993, when RVF

Table 3. Potential Rift Valley fever sylvatic vectors and viral isolates, Sénégal, 1991 to 1996

| Vector species | No. | No. pools | Virus isolates[a] (No. strains) |
|---|---|---|---|
| Barkedji | | | |
| *Aedes vexans* | 42,055 | 1,428 | WN-NRI (1), RVF (10), WN (2) |
| *Ae. mcintoshi* | 758 | 88 | NRI (1) |
| *Ae. ochraceus* | 3,672 | 228 | WSL (1), RVF (3) |
| *Ae. dalzieli* | 105 | 34 | (0 virus) |
| Phlebotominae spp. | 233,895 | 2,251 | SAB (63), CHP (7), GF (5), Ar D 88909 (1), Ar D 95737 (12) |
| Kédougou | | | |
| *Ae. vexans* | 1,194 | 81 | (0 virus) |
| *Ae. mcintoshi* | 536 | 107 | (0) virus) |
| *Ae. ochraceus* | 915 | 110 | WSL (1) |
| *Ae. dalzieli*[b] | 31,809 | 821 | CHIK (8), BBK (1), WSL (2), KED (6), BOU (1), PGA (2), ZIKA (22) |
| Phlebotominae spp. | 35,569 | 364 | SAB (11), CHP (4), TETE (1), Ar D 111740 (1), Ar D 95737 (2) |

[a]WN: West-Nile virus, NRI: Ngari virus, RVF: Rift Valley fever virus, WSL: Wesselsbron virus, SAB: Saboya virus, CHP: Chandipura virus, GF: Gabek Forest virus, CHIK: Chikungunya virus, BBK: Babanki virus, KED: Kédougou virus, BOU: Bouboui virus, PGA: Pongola virus, ZIKA: Zika virus, TETE: Tete virus. Ar D 88909, Ar D 95737, and Ar D 111740: not yet identified viruses.
[b]4 RVF strains were isolated in 1974 and 1982.

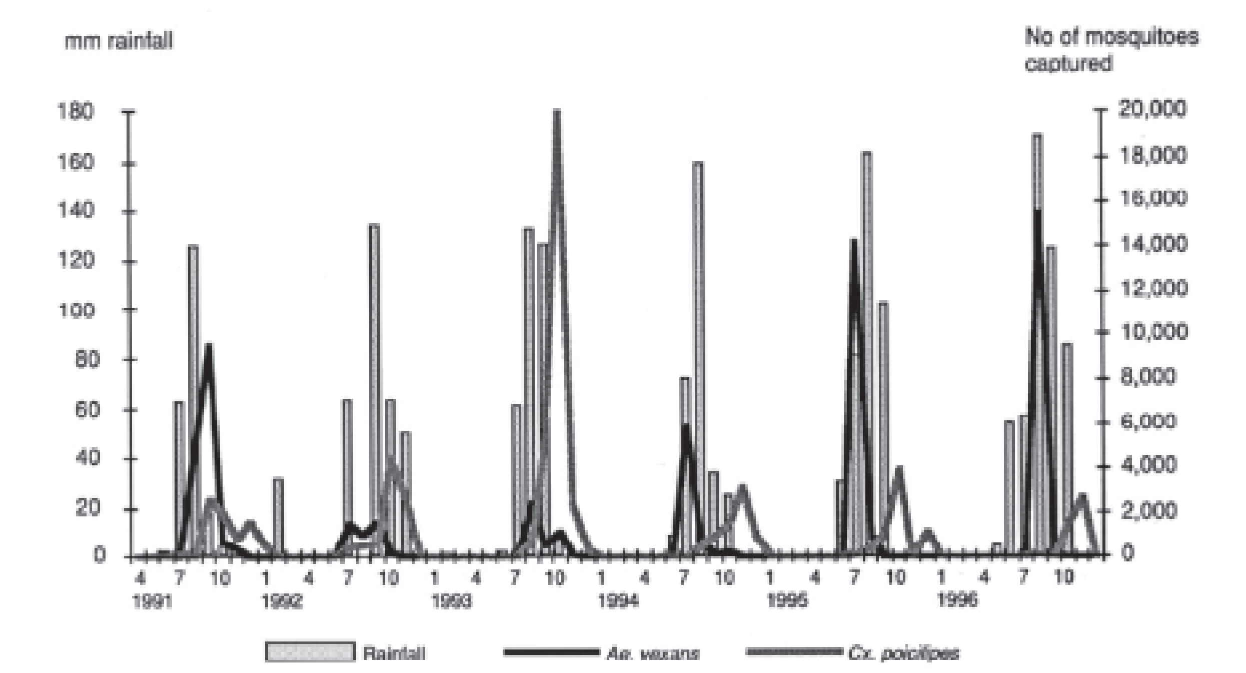

Figure. Distribution of *Aedes vexans* and *Culex poicilipes* captured by monthly rainfall, Barkedji, Sénégal, 1991-1996.

Table 4. Host choices of Rift Valley fever virus vectors captured with light traps or animal-bait traps in Sénégal, 1991-1996

| | No. of host-specific blood feedings | | | | |
|---|---|---|---|---|---|
| Vector | Cow | Sheep | Horse | Chicken | Human[a] |
| *Aedes vexans* | 35 | 33 | 38 | 7 | + + + |
| *Ae. ochraceus* | 8 | 18 | 5 | 1 | + + |
| *Ae. mcintoshi* | 7 | 20 | 2 | 2 | + + |
| *Ae. dalzieli* | 52 | 114 | 1 | 21 | + + + |

[a]++: numerous bites; +++: very numerous bites.

virus was isolated in Barkedji from floodwater *Aedes* and from one sheep, the situation was different. No environmental or climatic changes were identified; in particular, the rainfall was not higher than in previous years. However, a 15% IgM prevalence was observed in 1994 and 1995 among herds studied each year since 1990 along the Sénégal River, demonstrating RVF virus circulation. Enzootic vectors of RVF virus are now identified in West Africa, but factors causing outbreaks need to be further studied.

### Acknowledgments

We thank Leonard Munstermann, who organized the Emerging Vectors of Emergent Diseases symposium {sponsored by the American Commitee of Medical Entomology, held in Orlando during the XX meeting of the American Society of Tropical Medicine and Hygiene (7-11 December 1997), where these data were presented and Scott McKeown for help with the English translation.

This work was supported by the Institut Francais de Recherche Scientifique pour le Développement en coopération (ORSTOM) and by the Dakar Pasteur Institute.

### References

1. Meegan JM, Bailey CH. Rift Valley fever. In: Monath TP, editor. The arboviruses: epidemiology and ecology. Boca Raton (FL): CRC Press; 1988. p. 51-76.
2. Peters CJ. Emergence of Rift Valley fever. In: Saluzzo JF, Dodet B, editors. Factors in the emergence of arbovirus diseases. Paris: Elsevier; 1997. p.253-64.
3. World Health Organization. Emerging and other communicable diseases. Disease outbreaks reported, 28 January 1998: Rift Valley fever and haemorrhagic disease in Kenya and Somalia. [cited 1998 Mar 17]:[1 screen]. Available from URL: http://www.who.ch/emc/oldnews.htm.
4. Zeller HG, Fontenille D, Traore-Lamizana M, Thiongane Y, Digoutte JP. Enzootic activity of Rift Valley fever virus in Sénégal. Am J Trop Med Hyg 1997;56:265-72.
5. Zeller HG, Bessin R, Thiongane Y, Bapetel I, Teou K, Gbaguidi Ala M, Nde Atse A, Sylla R, Digoutte JP, Akakpo JA. Rift Valley fever antibody prevalence in domestic ungulates in several West-African countries (1989-1992) following the 1987 Mauritanian outbreak. Res Virol 1995;146:81-5.
6. Jouan A, Le Guenno B, Digoutte JP, Philippe B, Riou O, Adam F. A Rift Valley fever epidemic in Southern Mauritania. Ann Virol 1988;139:307-8.
7. Ksiazek TG, Jouan A, Meegan JM, Le Guenno B, Wilson ML, Peters CJ, Digoutte JP, Guillaud M, Merzoug NO, Touray EM. Rift Valley fever among domestic animals in the recent West African outbreak. Res Virol 1989;140:67-7.
8. Thiongane Y, Gonzalez JP, Fati A, Akakpo JA. Changes in Rift Valley fever neutralizing antibody prevalence among small domestic ruminants following the 1987 outbreak in the Sénégal river basin. Res Virol 1991;142:67-70.
9. Turell MJ, Perkins PV. Transmission of Rift Valley fever virus by the sand fly, *Phlebotomus duboscqi* (Diptera: Psychodidae). Am J Trop Med Hyg 1990;42:185-8
10. Fontenille D, Traore-Lamizana M, Zeller H, Mondo M, Diallo M, Digoutte JP. Rift Valley fever in Western Africa: isolations from *Aedes* mosquitoes during an interepizootic period. Am J Trop Med Hyg 1995;52:403-4.
11. Digoutte JP, Calvo-Wilson MA, Mondo M, Traore-Lamizana M, Adam F. Continuous cell lines and immune ascitic fluid pools in arbovirus detection. Res Virol 1992;143:417-22.
12. Beier JC, Perkins PV, Wirtz RA, Koros J, Diggs D, Gargam TPII, Koech DK. Bloodmeal identification by direct enzyme-linked immunosorbent assay (ELISA) tested on *Anopheles* (Diptera: Culicidae) in Kenya. J Med Entomol 1988;25:9-16.
13. Turrel MJ, Faran ME, Cornet M, Bailey CL. Vector competence of Sénégalese *Aedes fowleri* (Diptera: Culicidae) for Rift Valley fever virus. J Med Entomol 1988;25:262-6.
14. White GB. Note on a catalogue of *Culicidae* of the Ethiopian region. Mosquito Systematics 1975;7:303-44.
15. Horsfall WR, Novak RJ. The inland floodwater mosquito, *Aedes vexans:* an annotated bibliography to world literature. Pt I. J Am Mosq Control Assoc; 1991: 1-43.
16. Thonnon J. Rapport annuel 1995 du Centre Collaborateur OMS de Reference et de Recherche pour les Arbovirus et les virus de fievres hemorragiques (CRORA). Sénégal: Institut Pasteur de Dakar; 1996.
17. Saluzzo JF, Digoutte JP, Chartier C, Martinez D, Bada R. Focus of Rift Valley fever transmission in southern Mauritania. Lancet 1987;1:504.
